# A Sex-Specific Comparison of Major Depressive Disorder Symptomatology in the Canadian Forces and the General Population

**DOI:** 10.1177/070674371405900707

**Published:** 2014-07

**Authors:** Julie Erickson, D Jolene Kinley, James M Bolton, Mark A Zamorski, Murray W Enns, Jitender Sareen

**Affiliations:** 1 Student, Department of Psychology, University of Manitoba, Winnipeg, Manitoba.; 2 Assistant Professor, Department of Psychiatry, University of Manitoba, Winnipeg, Manitoba.; 3 Directorate of Mental Health, Canadian Forces Health Services Group Headquarters, Ottawa, Ontario.; 4 Professor, Departments of Psychiatry and Community Health Sciences, University of Manitoba, Winnipeg, Manitoba.; 5 Professor, Departments of Psychiatry, Psychology, and Community Health Sciences, University of Manitoba, Winnipeg, Manitoba.

**Keywords:** depression, major depressive disorder, symptomatology, symptom profiles, military, Canadian Forces, civilians, general population, sex

## Abstract

**Objective:**

To compare major depressive disorder (MDD) symptomatology within men and women in a large, representative sample of Canadian military personnel and civilians.

**Method:**

We used the Canadian Community Health Survey: Mental Health and Well-Being (Cycle 1.2 and Canadian Forces Supplement) (*n* = 36 984 and *n* = 8441, respectively) to compare past-year MDD symptomatology among military and civilian women, and military and civilian men. Logistic regression models were used to determine differences in the types of depressive symptoms endorsed in each group.

**Results:**

Men in the military with MDD were at lower odds than men in the general population to endorse numerous symptoms of depression, such as hopelessness (adjusted odds ratio [AOR] 0.44; 99% CI 0.23 to 0.83) and inability to cope (AOR 0.53; 99% CI 0.31 to 0.92). Military women with MDD were at lower odds of thinking about their death (AOR 0.52; 99% CI 0.32 to 0.86), relative to women with MDD in the general population.

**Conclusion:**

Different MDD symptomatology among males and females in the military, compared with those in the general population, may reflect selection effects (for example, personality characteristics and patterns of comorbidity) or occupational experiences unique to military personnel. Future research examining the mechanisms behind MDD symptomatology in military personnel and civilians is required.

## Abstract (fr)

**Objectif:**

Comparer la symptomatologie du trouble dépressif majeur (TDM) entre les hommes et les femmes d’un vaste échantillon représentatif du personnel militaire canadien et des civils.

**Méthode:**

Nous avons utilisé l’Enquête sur la santé dans les collectivités canadiennes— Santé mentale et bien-être (cycle 1.2 et le Supplément des Forces canadiennes) (*n* = 36 984 et *n* = 8441, respectivement) pour comparer la symptomatologie du TDM chez les femmes et les hommes militaires et civiles. Des modèles de régression logistique ont servi à déterminer les différences de types de symptômes dépressifs déclarés dans chaque groupe.

**Résultats:**

Les militaires hommes souffrant de TDM avaient moins de probabilités que ceux de la population générale de déclarer de nombreux symptômes de dépression, comme le désespoir (ratios de cotes corrigées [RCC] 0,44; IC à 99 % 0,23 à 0,83) et l’incapacité de s’adapter (RCC 0,53; IC à 99 % 0,31 à 0,92). Les femmes militaires souffrant de TDM avaient moins de probabilités de penser à leur mort (RCC 0,52; IC à 99 % 0,32 à 0,86), relativement aux femmes souffrant de TDM de la population générale.

**Conclusion:**

La symptomatologie du TDM différente chez les hommes et les femmes militaires, comparée à celle de ce et celles de la population générale, peut refléter des effets de sélection (par exemple, caractéristiques de la personnalité et modèles de comorbidité) ou des expériences professionnelles propres au personnel militaire. Il est nécessaire qu’une future recherche examine les mécanismes derrière la symptomatologie du TDM chez les militaires et les civils.

Major depressive disorder is a highly prevalent mental disorder that limits participation in society and lowers quality of life.^1^ A growing body of research has examined the extent to which MDD affects military personnel, particularly those who have experienced combat or other occupational stressors or traumatic events.^2^–^6^ A recent meta-analysis of epidemiologic studies of MDD in US military samples estimated a past-year prevalence of 12% to 13% for deployed personnel and 5.7% for never-deployed personnel.^2^ Data on the epidemiology and clinical characteristics of MDD (for example, episode length, impairment, and comorbidity) in Canadian military personnel are lacking, as are comparisons to the general population.

A sizable body of literature on the clinical characteristics of MDD indicates that symptomatology can be diverse, with different groups endorsing particular symptoms more often than others. Men and women differ significantly in terms of MDD symptom presentation, with women being more likely than men to report increased appetite and weight gain,^7^–^10^ sleep disturbances,^11^,^12^ fatigue,^13^,^14^ and psychomotor retardation.^11^,^13^–^15^ As such, it is reasonable to assert that men and women are distinct groups regarding MDD presentation. However, there may be additional differences in MDD symptomatology within particular subpopulations of men and women. Specifically, there is a noticeable lack of published research comparing how male and female military personnel differ from the general population in terms of the types of depressive symptoms endorsed. Information on MDD symptom profile differences in military personnel, compared with civilians, would be beneficial for clinicians to guide assessment and educational efforts and ultimately increase MDD recognition. Further, variability in symptom profiles may help shed light on differences in MDD prevalence between military personnel and the general population.

**Clinical Implications**

- Clinicians should be aware that when diagnosing MDD, military personnel may be at lower risk for endorsing several symptoms of MDD, such as hopelessness and inability to cope.
- Male military personnel with MDD may have a more restrained symptom presentation relative to men with MDD in the general population. Thorough assessment of MDD symptoms and comorbidity in military personnel is imperative.
- Despite female military personnel with MDD being at lower odds of thinking about their own death, ongoing assessment of suicidal ideation is still a critical component of diagnostic and treatment procedures.

**Limitations**

- The cross-sectional nature of the CCHS 1.2 and CCHS-CFS does not permit causal conclusions or any temporal relations.
- The CCHS 1.2 did not assess posttraumatic stress disorder or general anxiety disorder, which prohibited the inclusion of these mental disorders as a covariate.

The CCHS 1.2 and the CCHS-CFS provide a unique opportunity to conduct a sex-specific comparison of MDD symptoms in the military, compared with those in the general population (that is, military women compared with general population women, and military men compared with general population men). The CCHS 1.2 and the CCHS-CFS were conducted at similar time points, employed the same validated diagnostic instruments, and are representative of the general population and Canadian Forces personnel, respectively. As such, these surveys are ideal to compare MDD symptomatology among male and female military personnel and civilians.

## Methods

The CCHS 1.2 is a nationally representative survey of Canadians conducted in 2001–2002 by Statistics Canada that sought information about mental health and well-being. The sample consists of 36 984 people over the age of 15, with a response rate of 77%. The CCHS-CFS is a representative sample of active Canadian Forces personnel conducted by Statistics Canada and the Department of National Defence in 2002. The sample consists of 8441 people between the ages of 16 and 64, with 5155 regular force members and 3286 reserve force members. Response rates were 79.5% for regular force and 83.5% for reserve force. A multistage, stratified sampling technique was used for both the CCHS 1.2 and the CCHS-CFS to ensure that the samples were representative of the general population and Canadian military, respectively. The analyses were restricted to people with past-year MDD in both populations, yielding a sample size of *n* = 1944 in the general population sample and *n* = 560 in the military sample.

Both the general population and military survey used the World Mental Health Composite Interview Diagnostic Instrument to diagnose MDD according to the Diagnostic and Statistical Manual of Mental Disorders, Fourth Edition. Past-year diagnoses of MDD were assessed in both surveys using face-to-face interviewers. An extensive list of MDD symptoms were assessed, such as loss of interest in activities, sadness, hopelessness, discouragement, anhedonia, increased or decreased appetite, low energy, and psychomotor slowness. The general population and military survey gathered similar sociodemographic information, such as age, education, and marital status. The sociodemographic characteristics of both the general population^16^ and military^17^ samples have been documented elsewhere.

## Statistical Analysis

SUDAAN, version 11.0 (statistical software, Research Triangle Institute, Raleigh, NC), was used to complete the statistical analyses, with bootstrapping weights used for variance estimation. All analyses were restricted to people with a diagnosis of past-year MDD. Bivariate logistic regressions were also run for each depressive symptom to determine whether military men and women with past-year MDD differed from general population men and women with past-year MDD. All logistic regressions were adjusted for age, education, marital status, and comorbid mental disorders assessed in both surveys (that is, panic disorder, social phobia, and alcohol dependence), which have been correlated with MDD in previous research.^18^

## Results

Table 1 displays the prevalence of depressive symptoms in men with past-year MDD in the military and general population. Relative to the general population, men with past-year MDD in the military were significantly less likely to endorse experiencing hopelessness, psychomotor slowness, lost self-confidence, feeling not as good as others, inability to cope, and being in tears. The cell size for “sleeping more than usual” within the male military sample was too small to be released by Statistics Canada.

Corresponding results for women are shown in Table 2. Relative to the general population, women with past-year MDD in the military were at significantly lower odds of thinking about one’s death. The cell size for “sleeping more than usual” within the female military sample was too small to be released by Statistics Canada.

## Discussion

To our knowledge, our study was the first to use nationally representative data to conduct sex-specific comparisons of past-year MDD symptoms in the military, and the general population. Men in the military with past-year MDD were at lower odds than men in the general population to endorse numerous symptoms of depression, such as hopelessness, inability to cope, lost self-confidence, and feeling not as good as others. These trends suggest that MDD in military males is characterized by a more restrained symptom presentation; that is, this population is less prone to endorse ancillary symptoms of depression than men with MDD in the general population. Military women with past-year MDD were at lower odds of reporting an inability to cope and thinking about their death relative to those in the general population.

Explanations for the differences in depressive symptomatology between military and general population men and women likely involve a complex interplay between individual, social, and environmental factors. One possible explanation for these findings could stem from selection effects. Military personnel are not a random sample of people within the general population. These people are distinct in terms of personality characteristics^19^–^21^ and are situated within a particular occupational culture that could influence the expression and reporting of MDD symptoms. However, it is possible that other factors influencing depression may also be different within military personnel. Different contexts of help seeking and resources for mental health services^22^–^24^ for military personnel may influence symptomatology, such as coping beliefs and hope for the future, relative to people in the general population. Additionally, military personnel and civilians may also have patterns of mental disorder comorbidity^25^,^26^ that could conceivably influence MDD presentation in both of these populations.

Relative to men, there appeared to be more overlap in MDD symptomatology among women in the military and the general population. Nonetheless, the unique finding that military women with past-year MDD were at lower odds of thoughts about one’s death relative to women in the general population is likely a multifaceted phenomenon that warrants future consideration. It could be speculated that military service provides a sense of meaning or purpose for women with depression that influences the degree to which they think about their death. Future research to understand the experiences of female military personnel with MDD is necessary.

The data from the general population and military surveys are limited in that, first, they are cross-sectional. Longitudinal examinations of MDD symptoms in military personnel and civilians would be useful to shed light on how symptoms of MDD differ, at all, across time in both of these populations. Second, the general population survey did not assess posttraumatic stress disorder or general anxiety disorder, which did not permit the inclusion of these disorders as covariates in our analyses.

Additionally, low statistical power may be an issue within the military sample, given that analyses were restricted to people with past-year MDD and stratified by sex. Concurrently, there was a large number of statistical comparisons conducted, increasing the probability of type I error. We attempted to minimize the probability of type I error by using a more stringent 99% confidence interval. Nonetheless, the results here offer much-needed information on the symptom presentation of past-year MDD in men and women in the military and the general population.

Future research examining potential mechanisms behind these differences is needed. For example, research could explore the moderating role of personality characteristics or trauma exposure in MDD in military personnel, compared with civilians. Additional research could examine the functional impact of MDD on military personnel, compared with civilians, as a potential explanation for our observed differences in symptomatology. Nonetheless, in light of the findings summarized here, clinicians, particularly those who provide services to military personnel, may be better situated to assess and understand MDD in Canadian men and women. Further, symptom profile differences may shed light on observed differences in MDD prevalence rates among military personnel and the general population.

## Figures and Tables

**Table 1:** Depressive symptoms among men with past-year major depressive disorder in the military, compared with the general population

Symptom	Military %^a^	General population %^a^	AOR (99% CI)^b^
Discouraged about life	89.81	92.60	0.64 (0.22–1.82)
Loss of interest	86.73	80.86	1.17 (0.56–2.44)
Sadness every day	89.09	93.52	0.52 (0.19–1.44)
Hopelessness	61.65	78.05	0.44 (0.23–0.83)^c^
Lack of pleasure	75.27	79.24	0.75 (0.39–1.41)
Decreased appetite	62.67	63.97	0.75 (0.41–1.35)
Increased appetite	39.38	31.84	1.20 (0.43–3.37)
Gained weight	44.49	36.14	1.27 (0.47–3.48)
Lost weight	49.91	58.43	0.61 (0.34–1.09)
Slept more	[Table-fn tfn1-cjp-2014-vol59-july-393-398]—	55.17	[Table-fn tfn1-cjp-2014-vol59-july-393-398]—
Slept less	49.94	51.15	1.02 (0.57–1.82)
Low energy	86.60	90.22	0.69 (0.31–1.51)
Psychomotor slowness	49.57	64.34	0.48 (0.27–0.83)^c^
Restless–jittery	44.70	42.25	1.12 (0.47–2.70)
Thoughts came slowly	72.01	75.67	0.85 (0.45–1.60)
Thoughts racing	57.10	38.44	2.24 (0.67–7.57)
Difficulty concentrating	89.88	89.89	0.97 (0.39–2.42)
Difficulty making decisions	70.30	73.51	0.82 (0.45–1.49)
Lost self-confidence	65.75	83.46	0.35 (0.18–0.69)^c^
Felt not as good as others	53.14	67.56	0.56 (0.32–0.99)^d^
Worthlessness	56.31	62.27	0.64 (0.31–1.29)
Guilt	51.66	58.23	0.80 (0.45–1.42)
Irritability	76.75	69.12	1.09 (0.60–1.97)
Could not cope	48.09	65.28	0.53 (0.31–0.92)^c^
Wanting to be alone	84.64	81.28	1.39 (0.64–3.01)
Less talkative	88.17	87.34	1.15 (0.52–2.55)
Often in tears	32.97	45.10	0.56 (0.32–0.98)^d^
Thought about one’s death	52.30	63.95	0.85 (0.49–1.44)
Thought it would be better if dead	36.57	50.28	0.77 (0.44–1.37)

— = cell sizes too small to permit the release of percentages from Statistics Canada; AOR = adjusted odds ratio

a Weighted percentages are reported, as the cell sizes were too small to permit Statistics Canada to release the unweighted ns from the Canadian Community Health Survey, Canadian Forces Supplement without compromising anonymity of respondents.

b General population is the reference population

c *P* < 0.001;

d *P* < 0.01

**Table 2:** Depressive symptoms among women with past-year major depressive disorder in the military, compared with the general population

Symptom	Military %^a^	General population %^a^	AOR (99% CI)^b^
Discouraged about life	89.34	89.87	0.94 (0.37–2.38)
Loss of interest	89.74	86.39	1.81 (0.81–4.00)
Sadness every day	94.48	94.94	1.26 (0.45–3.59)
Hopelessness	70.29	74.65	1.08 (0.61–1.89)
Lack of pleasure	76.38	77.89	0.95 (0.53–1.73)
Decreased appetite	56.81	66.07	0.65 (0.38–1.10)
Increased appetite	64.12	48.65	1.61 (0.70–3.73)
Gained weight	61.04	49.84	1.21 (0.55–2.66)
Lost weight	65.12	64.79	0.78 (0.43–1.44)
Slept more	[Table-fn tfn6-cjp-2014-vol59-july-393-398]—	67.14	[Table-fn tfn6-cjp-2014-vol59-july-393-398]—
Slept less	50.19	44.86	1.27 (0.76–2.13)
Low energy	92.63	91.65	1.62 (0.62–4.26)
Psychomotor slowness	55.69	64.08	0.71 (0.43–1.19)
Restless–jittery	24.25	31.62	0.59 (0.23–1.48)
Thoughts came slowly	73.83	73.33	0.96 (0.58–1.60)
Thoughts racing	44.50	37.56	1.29 (0.48–3.50)
Difficulty concentrating	92.13	88.26	1.64 (0.67–3.99)
Difficulty making decisions	70.15	76.87	0.73 (0.43–1.21)
Lost self-confidence	85.31	79.86	1.61 (0.86–3.00)
Felt not as good as others	69.02	68.50	1.13 (0.65–1.93)
Worthlessness	64.83	76.60	0.64 (0.34–1.20)
Guilt	62.55	58.81	1.04 (0.66–1.66)
Irritability	71.98	66.86	1.06 (0.63–1.79)
Could not cope	54.90	66.06	0.69 (0.42–1.14)
Wanting to be alone	83.09	83.45	0.97 (0.52–1.82)
Less talkative	83.01	85.85	0.73 (0.38–1.42)
Often in tears	78.79	82.42	0.83 (0.46–1.53)
Thought about one’s death	49.69	69.68	0.52 (0.32–0.86)^c^
Thought it would be better if dead	43.68	49.75	0.80 (0.49–1.30)

— = cell sizes too small to permit the release of percentages from Statistics Canada; AOR = adjusted odds ratio

a Weighted percentages are reported, as the cell sizes were too small to permit Statistics Canada to release the unweighted ns from the Canadian Community Health Survey, Canadian Forces Supplement without compromising anonymity of respondents.

b General population is the reference population

c *P* < 0.001

## Abbreviations

AOR: adjusted odds ratio
CCHS: 1.2 Canadian Community Health Survey: Mental Health and Well-Being
CCHS-CFS: Canadian Community Health Survey, Canadian Forces Supplement
MDD: major depressive disorder
